# Supplementary material for: c-di-GMP inhibits the DNA binding activity of H-NS in Salmonella
Source: Nat Commun. 2023 Nov 18;14:7502. doi: 10.1038/s41467-023-43442-5 (PMC10657408; doi:10.1038/s41467-023-43442-5)
Supplement: Supplementary file 1 — Supplementary Information [file 41467_2023_43442_MOESM1_ESM.pdf]

Supplementary Information

c-di-GMP inhibits the DNA binding activity of H-NS in *Salmonella*

Shuyu Li[1], Qinmeng Liu[1], Chongyi Duan[1], Jialin Li[1], Hengxi Sun[1], Lei Xu[1], Qiao Yang[2,3], Yao Wang[1], Xihui Shen[1]*, Lei Zhang[1]*

[1]State Key Laboratory of Crop Stress Biology for Arid Areas, Shaanxi Key Laboratory of Agricultural and Environmental Microbiology, College of Life Sciences, Northwest A&F University, Yangling, Shaanxi 712100, China.

[2]ABI Group, College of Marine Science and Technology, Zhejiang Ocean University, Zhoushan 316021, China.

[3]Donghai Laboratory, Zhoushan 316021, China.

*For correspondence:

Xihui Shen (xihuishen@nwsuaf.edu.cn);

Lei Zhang (zhanglei0075@nwsuaf.edu.cn).

This PDF file includes:

Supplementary Figures 1-17

Supplementary Tables 1-2

Supplementary References

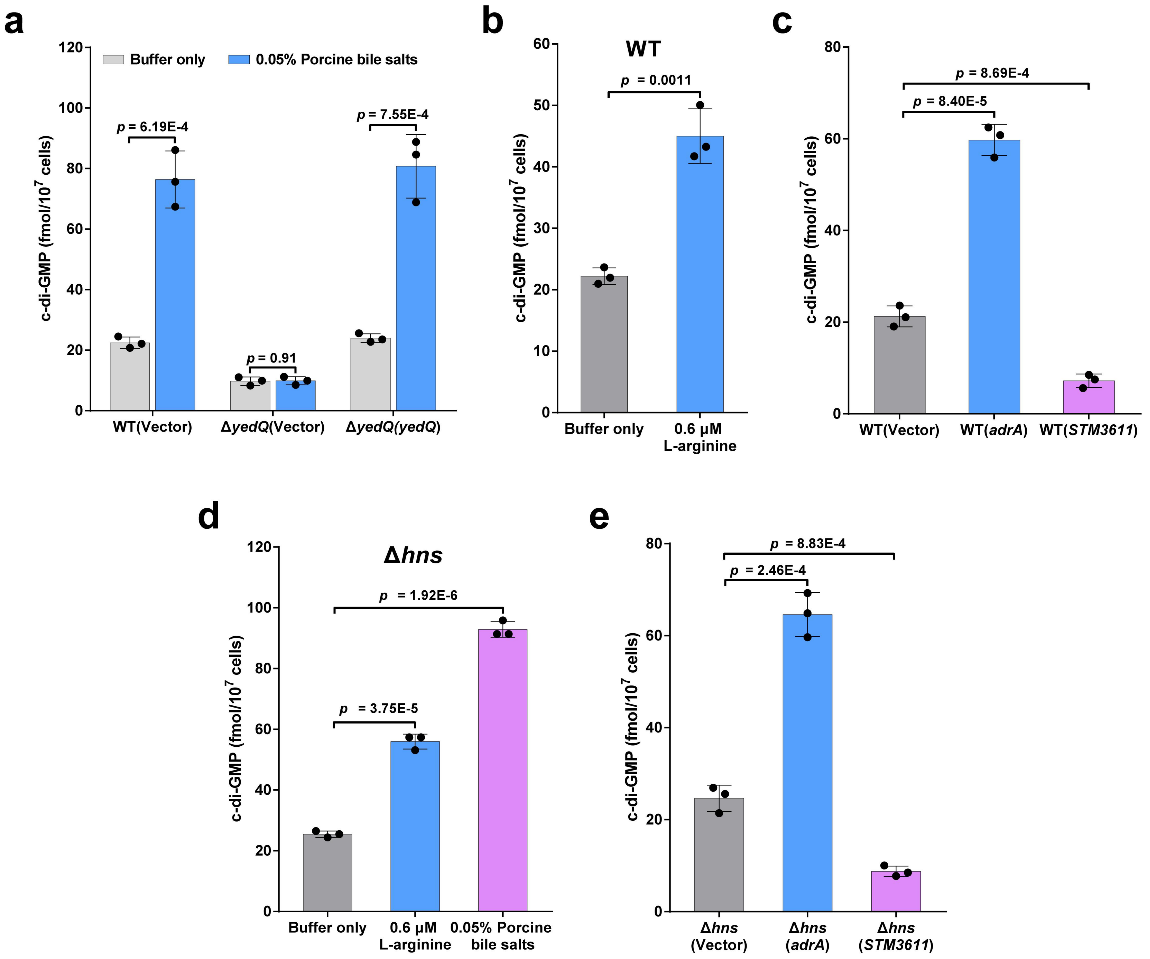

Supplementary Fig. 1 Intracellular c-di-GMP levels in *S*. Typhimurium are changed by stimulation with L-arginine and bile salts, or by overexpression of *adrA* and *STM3611*.

a An increase in intracellular c-di-GMP concentrations in response to bile salts requires YedQ. **b** L-Arginine stimulates an increase in intracellular c-di-GMP concentrations in the wild type (WT). **c** Intracellular c-di-GMP levels in the wild type were modulated by overexpression of *adrA* or *STM3611*. **d** L-Arginine and bile salts stimulate an increase in intracellular c-di-GMP concentrations in the Δ*hns* mutant. **e** Intracellular c-di-GMP levels in the Δ*hns* mutant were modulated by overexpression of *adrA* or *STM3611*. Data shown are mean ± SD of three biological replicates. Statistical significance was calculated using the two-tailed unpaired Student's *t*-test, and $p < 0.05$ was considered to be statistically significant. Source data are provided as a Source Data file.

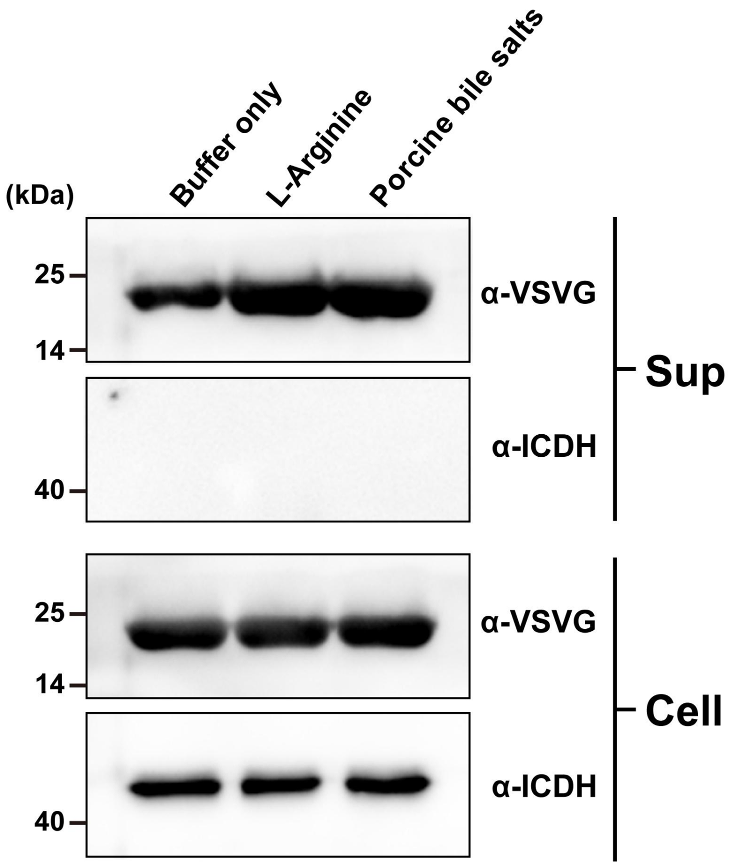

Supplementary Fig. 2. L-Arginine and bile salts stimulate Hcp1 secretion in *S.* Typhimurium SL1344.

S. Typhimurium SL1344 carrying a plasmid expressing Hcp1-VSVG were induced by 0.6 μM L-arginine, 0.05% bile salts or a buffer control, and then Hcp1-VSVG in cell pellet (Cell) and concentrated supernatant (Sup) was analyzed by western blot. ICDH was used as a loading control. Data shown were one representative from three independent experiments with similar results. Source data are provided as a Source Data file.

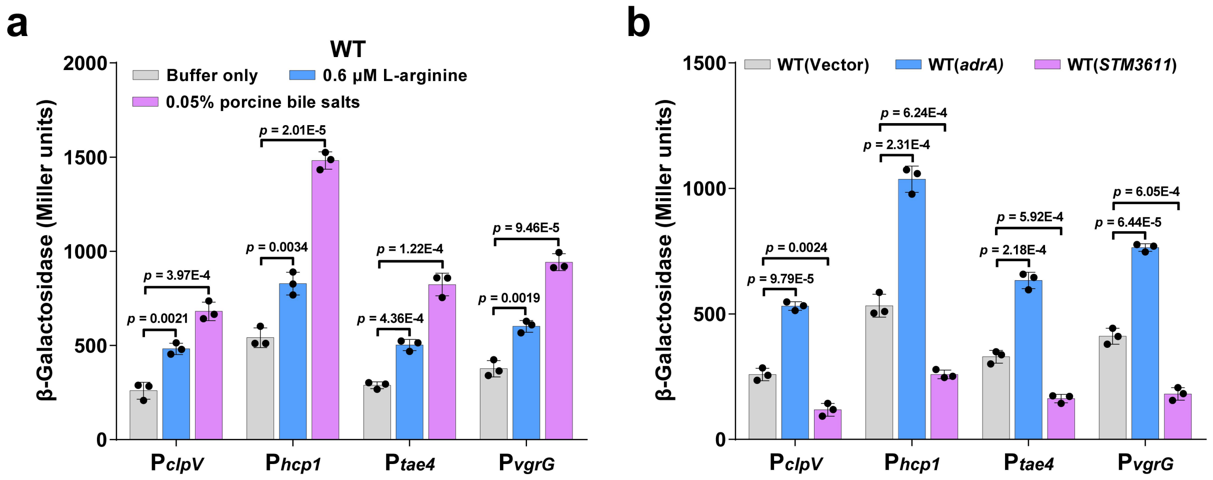

Supplementary Fig. 3. Elevated c-di-GMP levels induce the promoter activities of T6SS genes in *S*. Typhimurium SL1344.

a The promoter activities of *clpV*, *hcp1*, *tae4* and *vgrG* in the wild-type strain were induced by L-arginine or bile salts. **b** The promoter activities of the T6SS genes in the wild type (WT) were upregulated by overexpressing *adrA*, but downregulated by overexpressing *STM3611*. Data shown are mean ± SD of three biological replicates. Statistical significance was calculated using the two-tailed unpaired Student's *t*-test. *P* values < 0.05 indicate significant differences. Source data are provided as a Source Data file.

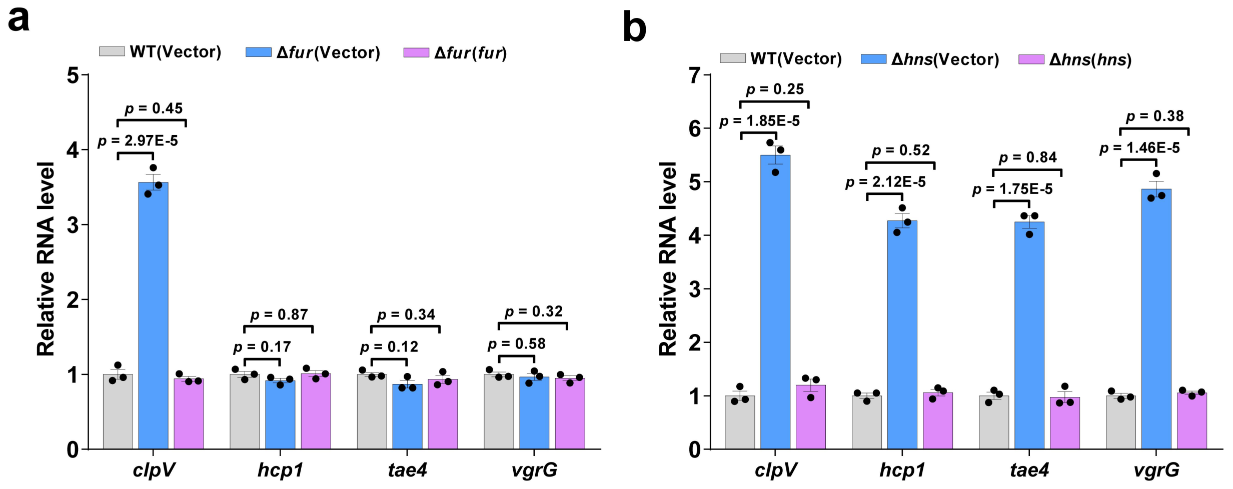

Supplementary Fig. 4. T6SS gene expression in *S*. Typhimurium wild type, mutants, and the corresponding complemented strains.

a qRT-PCR analysis of T6SS gene expression in *S.* Typhimurium wild type (WT), the Δ*fur* mutant and its complemented strain. **b** qRT-PCR analysis of T6SS gene expression in *S.* Typhimurium wild type, the Δ*hns* mutant and its complemented strain. Gene expression levels were normalized to 16S rRNA and presented as values relative to that of the wild type. Data are mean ± SD of three biological replicates. *P* values < 0.05 indicate significant differences (two-tailed unpaired Student's *t* test). Source data are provided as a Source Data file.

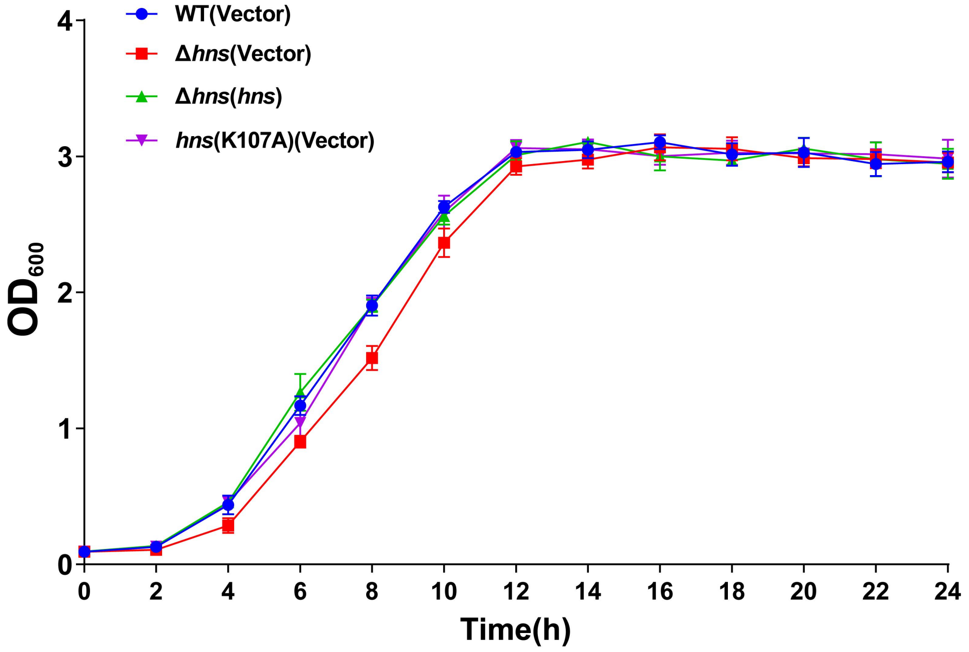

Supplementary Fig. 5. Growth curves of *S*. Typhimurium strains.

S. Typhimurium strains were grown in LB medium with shaking at 37 °C. 0.5 mM IPTG was added as an inducer at the time of inoculation. At each time point during growth, cell density was recorded by measuring the optical density at 600 nm. Data are mean ± SD of three biological replicates. WT, wild type. Source data are provided as a Source Data file.

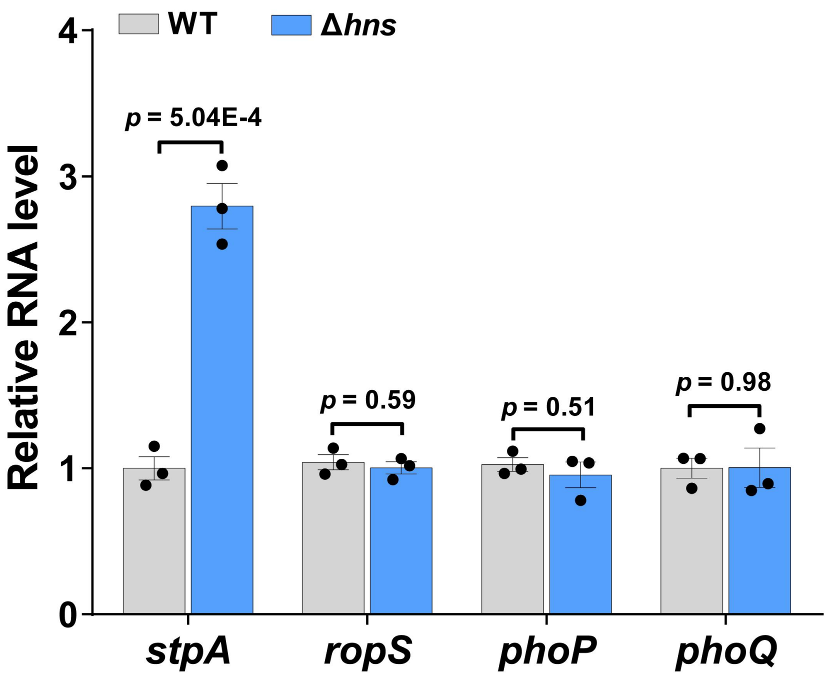

Supplementary Fig. 6. Expression of *stpA*, but not *rpoS* or the *phoPQ* operon, was altered in Δ*hns* compared to the wild type.

qRT-PCR analysis of gene expression in *S.* Typhimurium wild type (WT) and the Δ*hns* mutant. Gene expression levels were normalized to 16S rRNA and presented as values relative to that of the wild type. Data are mean ± SD of three biological replicates. $P < 0.05$ indicates that the difference is statistically significant (two-tailed unpaired Student's *t*-test). Source data are provided as a Source Data file.

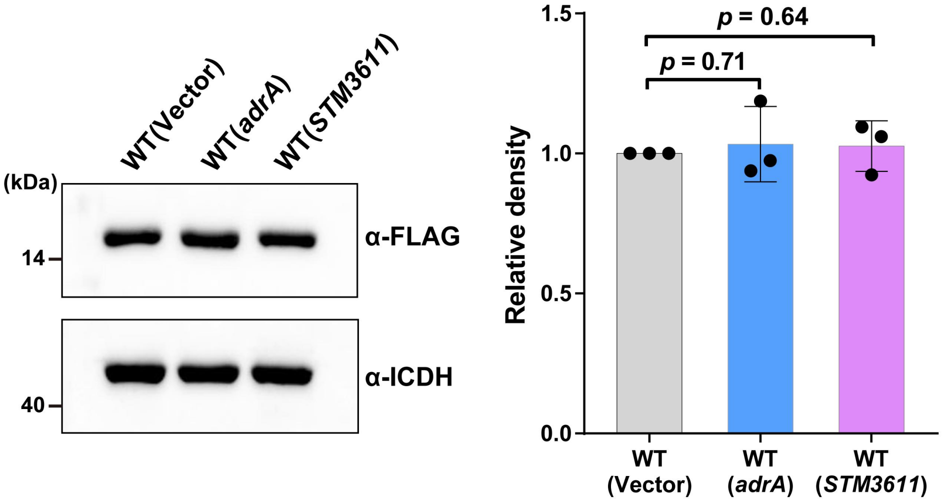

Supplementary Fig. 7. The production of H-NS in *S.* Typhimurium wild type is not affected by overexpression of *adrA* or *STM3611*.

The plasmid pBBR1MCS1 and its derivatives harboring *adrA* or *STM3611* were separately transformed into the wild-type (WT) strain expressing *in situ* tagged FLAG-H-NS, and the production of H-NS in cells were assessed by western blot analysis with anti-FLAG antibody. The blots shown are representative of three independent experiments with similar results. The band intensities were quantified by scanning densitometry using ImageJ (NIH, USA), normalized to intracellular ICDH, and presented as values relative to that of the wild type without gene overexpression (mean ± SD; n = 3 independent experiments). Differences were considered to be statistically significant when p values were less than 0.05 (two-tailed unpaired Student's t-test). Source data are provided as a Source Data file.

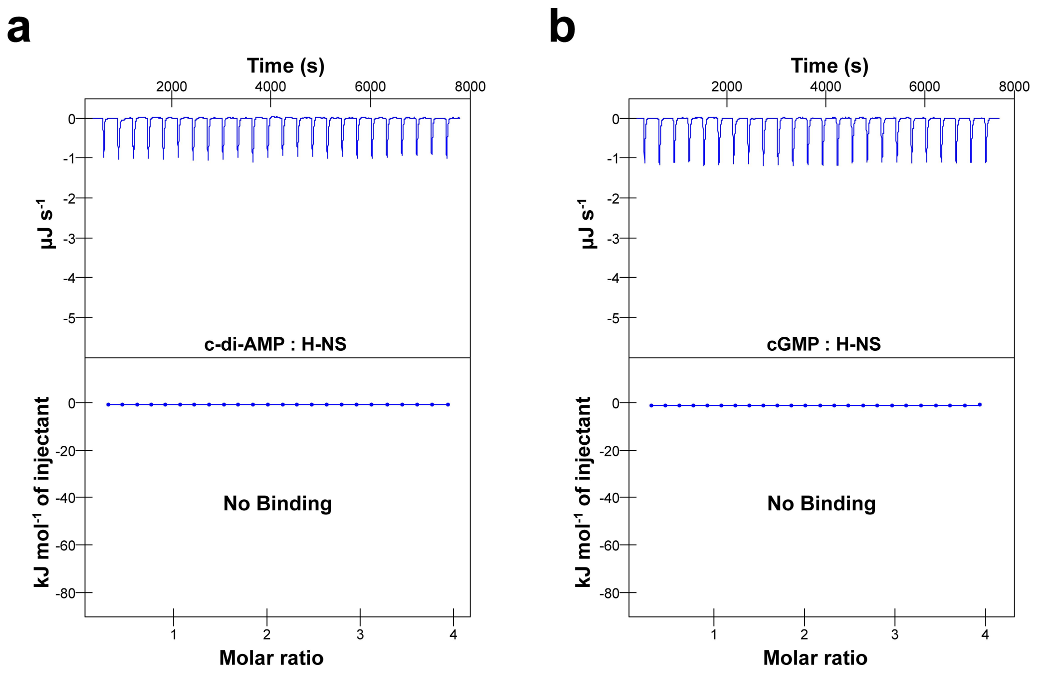

Supplementary Fig. 8. Isotherms representing binding of the H-NS protein of *S.* Typhimurium with c-di-AMP or cGMP, as measured by ITC.

a, b ITC data and plots of injected heat for injections of 100 μM c-di-AMP (**a**) or cGMP (**b**) into the sample cell containing 10 μM H-NS are shown in the upper and lower plots, respectively. The heats of ligand dilution were subtracted and the corrected data were fit to a one-site binding model by using the NanoAnalyze software. Isotherms shown are one representative of three independent experiments with similar results.

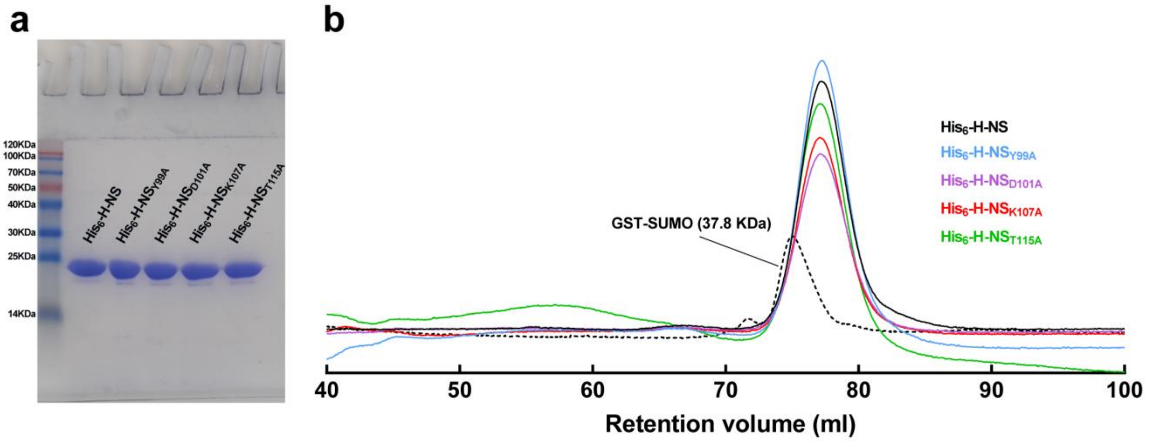

Supplementary Fig. 9. SDS-PAGE and SEC elution profiles of H-NS and its variants.
a An SDS-PAGE gel displaying purified H-NS and its variants. H-NS and its variants with an N-terminal His_6 tag were purified by Ni^{2+}-NTA affinity chromatography and then subjected to SDS-PAGE analysis. Similar results were obtained in three independent experiments. **b** SEC elution profiles of H-NS and its variants. After being concentrated, the purified H-NS and its variants were subjected to SEC using an ÄKTA Purifier FPLC system. The GST-SUMO fusion protein was used as the molecular weight standards. SEC analysis showed that purified recombinant H-NS and its variants had formed homodimers in solution. Data reproduced in three independent experiments. Source data are provided as a Source Data file.

a

**H-NS**	**91**	AARPAKYSYVDENGETKTWTGQGRTPAVIKKAMEE	**125**
**StpA**	**90**	QPRPAKYRFTDFNGEEKTWTGQGRTPKPIAQALAA	**124**

b

Binding affinity for c-di-GMP

**StpA**	**K_d (μM)**
WT	73 ± 14
F98Y	0.64 ± 0.09

Supplementary Fig. 10. StpA is a closely-related paralogue of H-NS but shows very low binding affinity for c-di-GMP.

a Sequence alignment of H-NS and its paralogue StpA in *S*. Typhimurium. Four key residues of H-NS involved in interactions with c-di-GMP are highlighted in yellow. The three conserved residues of StpA corresponding to D101, K107 and T115 of H-NS are highlighted in yellow, and the non-conserved residue corresponding to Y99 is highlighted in purple. **b** Binding of c-di-GMP to wild-type (WT) StpA and the F98Y variant. The binding affinity was evaluated by ITC analysis. The K_d values are presented as mean ± SD of three independent experiments. Source data are provided as a Source Data file.

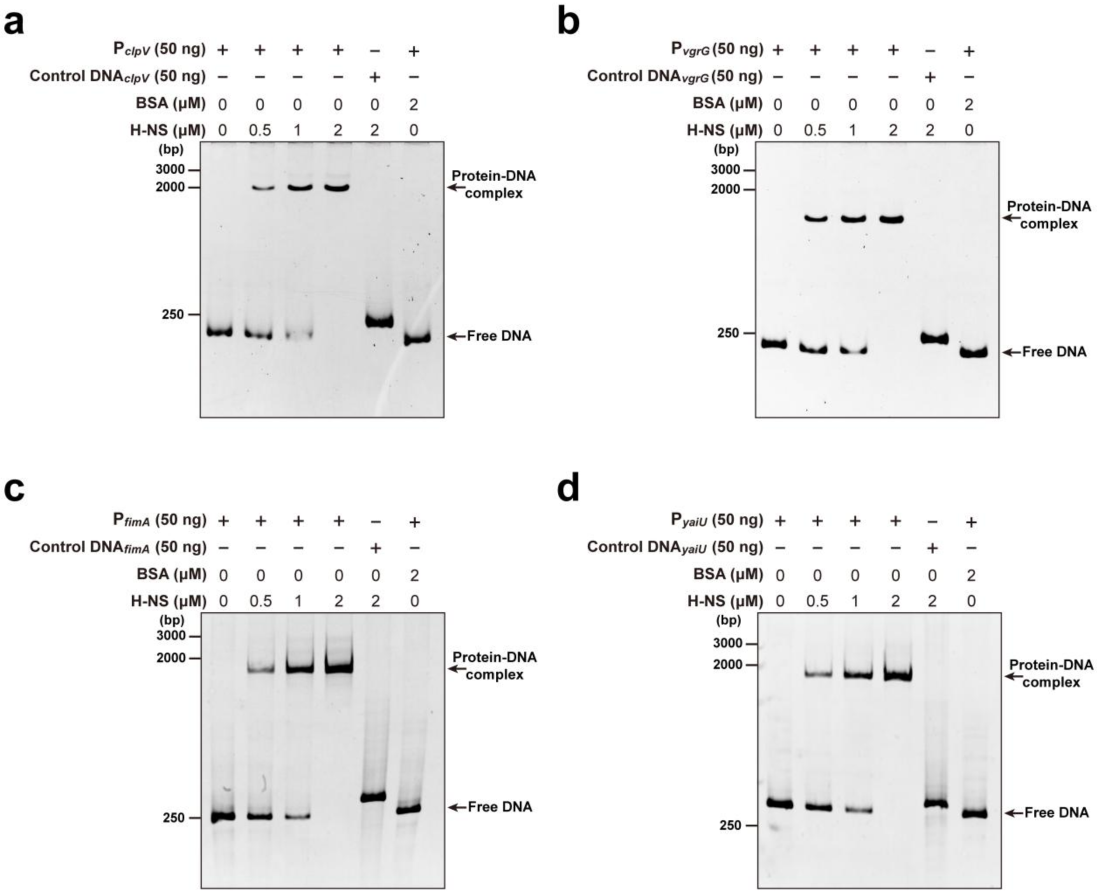

Supplementary Fig. 11. EMSAs demonstrating specificity of H-NS binding to promoters of *clpV*, *vgrG*, *fimA* and *yaiU*.

a-d 50 ng of DNA of P_{clpV} (**a**), P_{vgrG} (**b**), P_{fimA} (**c**) or P_{yaiU} (**d**) (the average AT content of each promoter sequence is above 57.9%) was incubated with H-NS (the concentrations are noted in the panel) in a 20-μl reaction system. DNA fragments amplified from the coding regions of the corresponding genes were used as DNA negative controls (the average AT content of each control sequence is below 43.6%) and BSA was used as the protein control. Gels shown are one representative of three independent experiments with similar results. Source data are provided as a Source Data file.

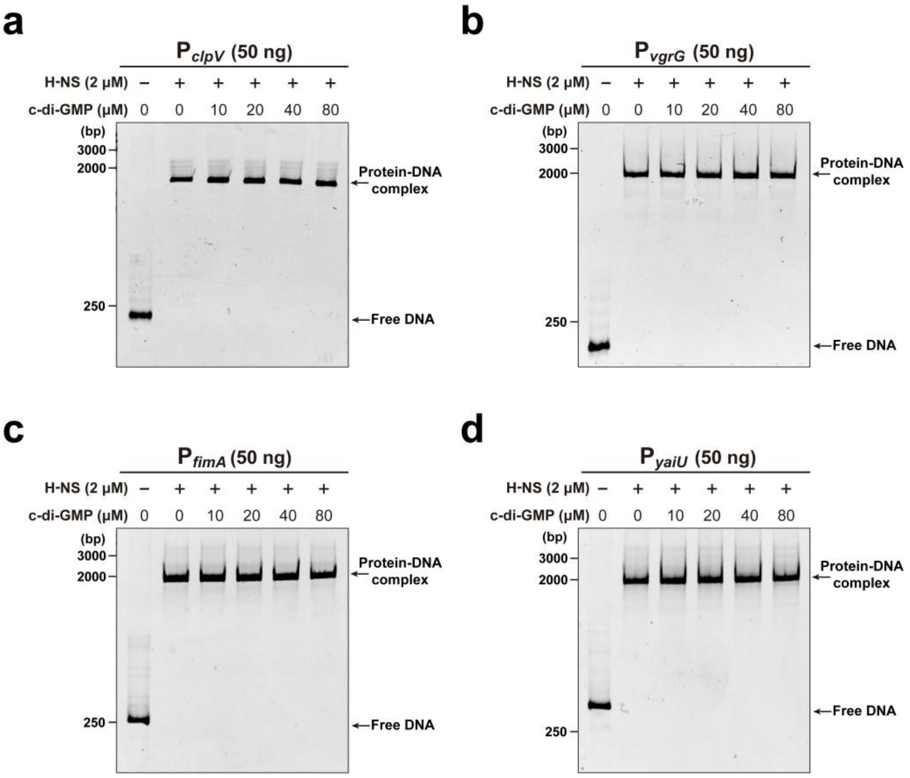

Supplementary Fig. 12. c-di-GMP has no ability to displace H-NS from DNA.

a-d DNA of P_{clpV} (**a**), P_{vgrG} (**b**), P_{fimA} (**c**) or P_{yaiU} (**d**) was first incubated with H-NS for 20 min, followed by the subsequent addition of c-di-GMP at the indicated concentrations and incubation for another 20 min. Gels shown are one representative of three independent experiments with similar results. Source data are provided as a Source Data file.

a

Binding affinity for FLAG–H-NS

Nucleotides/ DNA probes	K_d (μM)
c-di-GMP	0.29 ± 0.06
P_{clpV}	0.36 ± 0.04
P_{vgrG}	0.48 ± 0.08
P_{fimA}	0.32 ± 0.06
P_{yaiU}	0.31 ± 0.09

b

Binding affinity for $H\text{-}NS_{T115A}$

DNA probes	K_d (μM)
P_{clpV}	27 ± 8
P_{vgrG}	18 ± 5

Supplementary Fig. 13. FLAG-H-NS maintains unaffected c-di-GMP- and DNA-binding activity while $H\text{-}NS_{T115A}$ showed dramatically reduced DNA-binding activity.

a Binding of FLAG-H-NS for c-di-GMP or its target gene promoters. **b** Binding of $H\text{-}NS_{T115A}$ for the promoter sequences of *clpV* and *vgrG*. The binding affinity was measured by ITC, and the K_d values were presented as mean ± SD of three independent experiments. Source data are provided as a Source Data file.

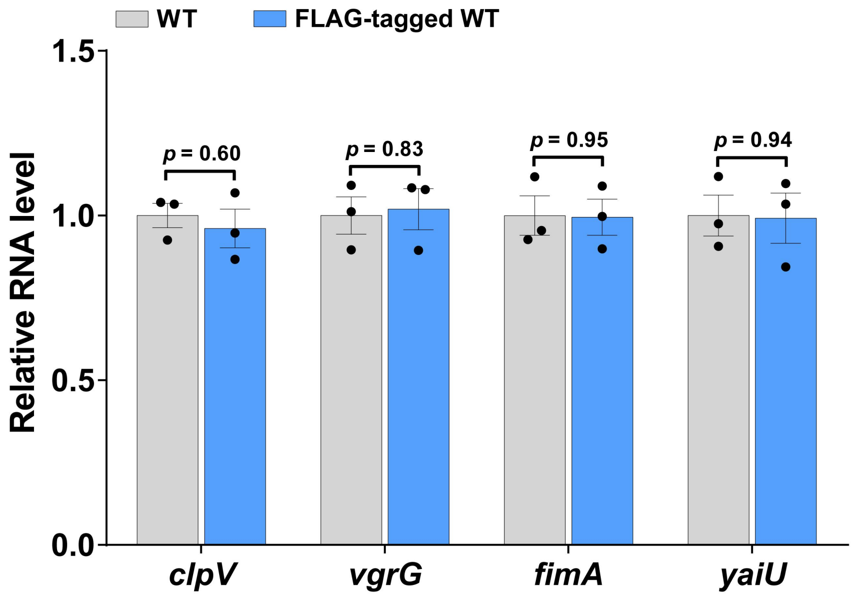

Supplementary Fig. 14. The mRNA levels of the four H-NS-repressed genes are not altered by the presence of the FLAG tag.

qRT-PCR analysis of the expression of the four H-NS-repressed genes in FLAG-tagged and untagged wild-type (WT) *S*. Typhimurium. Gene expression levels were normalized to 16S rRNA and presented as values relative to that of the untagged wild type. Data are mean ± SD of three biological replicates. Statistical significance was calculated using the two-tailed unpaired Student's *t*-test, and differences were considered to be statistically significant when *p* values were less than 0.05. Source data are provided as a Source Data file.

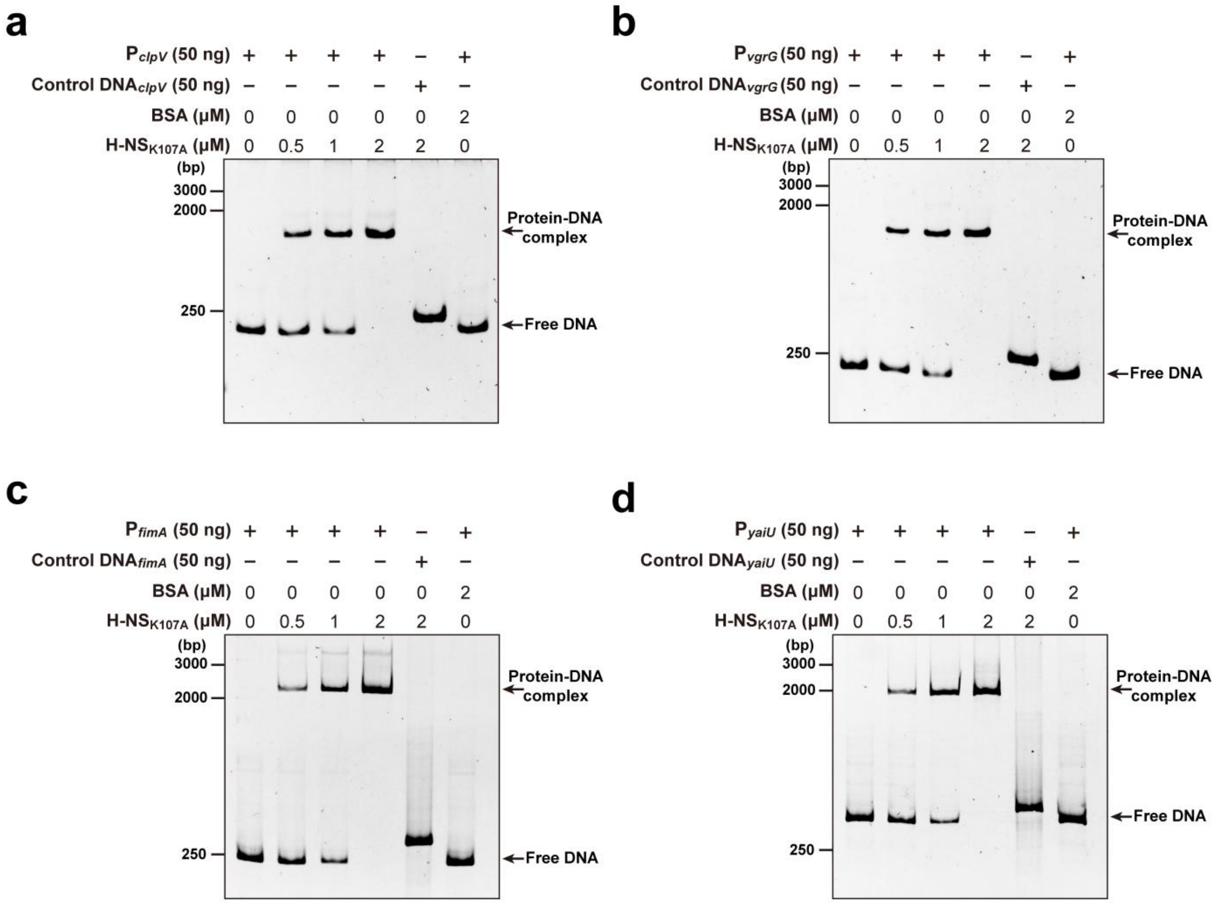

Supplementary Fig. 15. EMSAs demonstrating specificity of H-NS$_{K107A}$ binding to promoters of *clpV*, *vgrG*, *fimA* and *yaiU*.

a-d 50 ng of DNA of P$_{clpV}$ (**a**), P$_{vgrG}$ (**b**), P$_{fimA}$ (**c**) or P$_{yaiU}$ (**d**) was incubated with H-NS$_{K107A}$ (the concentrations are noted in the panel) in a 20-μl reaction system. DNA fragments amplified from the coding regions of the corresponding genes were used as DNA negative controls and BSA was used as the protein control. Gels shown are one representative of three independent experiments with similar results. Source data are provided as a Source Data file.

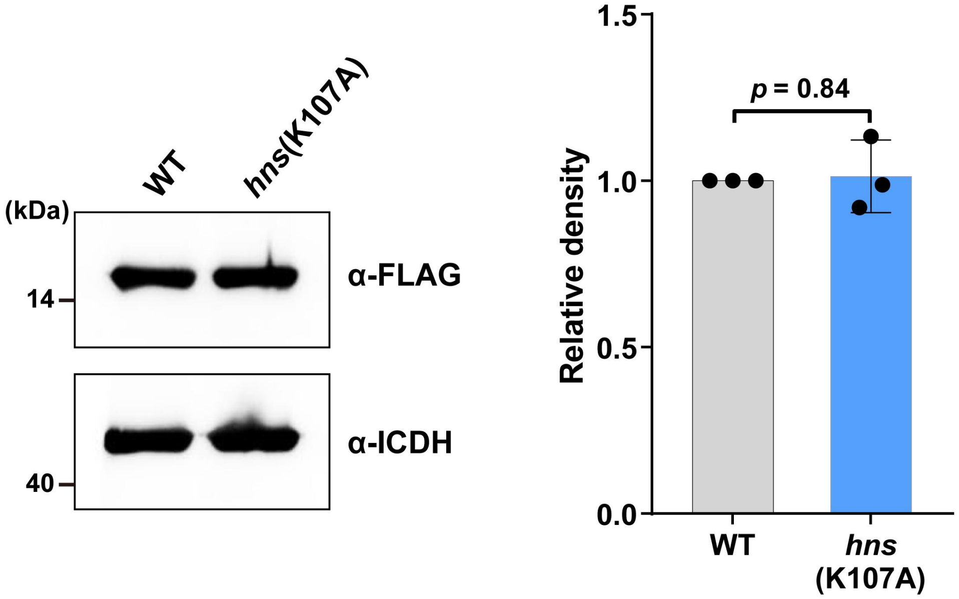

Supplementary Fig. 16. The K107A substitution in H-NS did not affect the abundance of the protein in *S.* Typhimurium.

Western blot analysis of intracellular accumulation of H-NS and H-NS_{K107A} in the wild type (WT) and the *hns*(K107A) mutant with a FLAG tag fused to the N terminus of the *hns/hns*(K107A) gene in the chromosome. The blots shown are representative of three independent experiments with similar results. The band intensities were quantified by scanning densitometry using ImageJ (NIH, USA), normalized to intracellular ICDH, and presented as values relative to that of the wild type (mean ± SD; n = 3 independent experiments). Differences were considered to be statistically significant when p values were less than 0.05 (two-tailed unpaired Student's t-test). Source data are provided as a Source Data file.

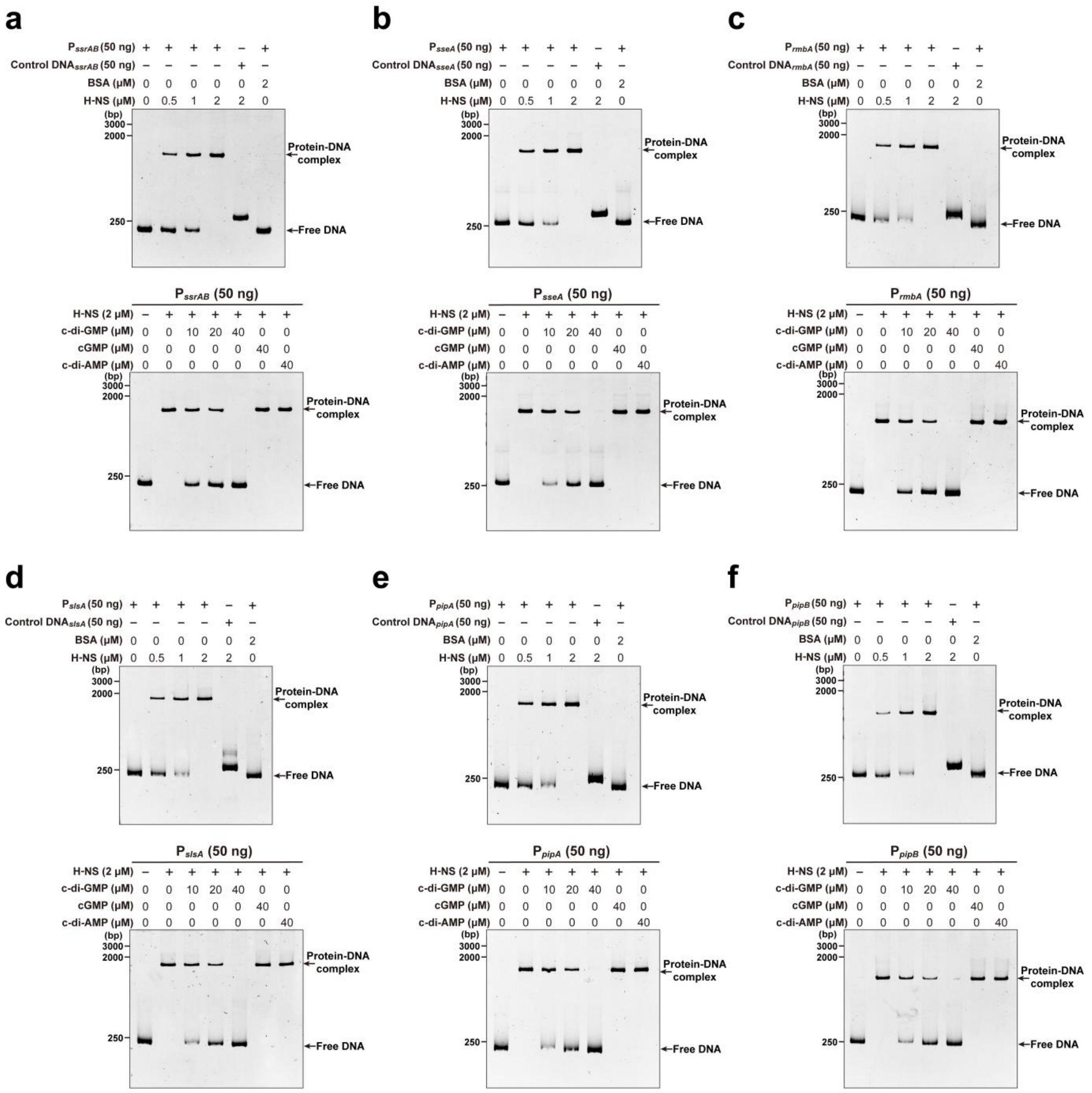

Supplementary Fig. 17. Specific binding of H-NS to the promoters of the target genes or operons within SPI-2, SPI-3 and SPI-5 is inhibited by c-di-GMP.

a-f EMSA assays show that H-NS specifically binds to the promoter sequences of *ssrAB* (**a**), *sseA* (**b**), *rmbA* (**c**), *slsA* (**d**), *pipA* (**e**) or *pipB* (**f**) (top), and the binding is impaired by c-di-GMP in a dose-dependent fashion (bottom). DNA fragments amplified from the coding regions of the corresponding genes or operons were used as DNA negative controls and BSA was used as the protein control (**a-f**, top). The average AT content of each promoter sequence is above 56.1%, while the average AT content of each DNA negative control is below 44.8%. Gels shown are one representative of three independent experiments with similar results. Source data are provided as a Source Data file.

Supplementary Tables

Table S1. Bacterial strains and plasmids used in this study.

Strains and plasmids	Relevant characteristics*	Source
**Strains**		
***S.* Typhimurium**		
SL1344	Wild-type	Laboratory stock
Δ*yedQ*	*yedQ* deletion mutant in SL1344	1
Δ*fur*	*fur* deletion mutant in SL1344	This study
Δ*hns*	*hns* deletion mutant in SL1344	This study
Δ*clpV*	*clpV* double deletion mutant in SL1344	This study
hns(K107A)	SL1344 Wild-type strain with the K107A mutation *of hns*	This study
FLAG-*hns*	SL1344 Wild-type strain expressing in *situ* tagged FLAG–H-NS	This study
FLAG-hns_{K107A}	SL1344 Wild-type strain expressing in *situ* tagged FLAG–H-NS_{K107A}	This study
E. coli		
BL21(DE3)	Host for expression vector pET-28a	Novagen
TG1	Host for cloning	Stratagene
S17-1λpir	λ-pir lysogen of S17-1, F^- *thi pro hsdR* [RP4-2 Tc::Mu Km::Tn7 (Tp Sm)]	2
**Plasmids**		
pCas	Crispr-Cas9 system plasmid used for in-frame deletion, Km^r	3
pTargetF1	pTargetF with the spectinomycin resistance gene replaced by a chloramphenicol resistance gene, Cm^r	4
pTargetF1-Δ*yedQ*	pTargetF1 derivative for *yedQ* deletion in SL1344	This study
pTargetF1-Δ*fur*	pTargetF1 derivative for *fur* deletion in SL1344	This study
pTargetF1-Δ*hns*	pTargetF1 derivative for *hns* deletion in SL1344	This study
pTargetF1-Δ*clpV*	pTargetF1 derivative for *clpV* deletion in SL1344	This study
pTargetF1-*hns*(K107A)	pTargetF1 derivative for the K107A mutation *of hns* in SL1344	This study
pTargetF1–FLAG-*hns*	pTargetF1 derivative for in *situ* tagged FLAG–H-NS and FLAG–H-NS_{K107A} in SL1344	This study
pDM4	Suicide vector, *mob*RK2, *ori*R6K, *pir*, *sacB*, Cm^r	6
pDM4-*lacZ*	pDM4 derivative with a promoterless *lacZ* gene inserted into the multiple cloning site	1
pDM4-P_{clpV}::*lacZ*	pDM4 derivative for *clpV* promoter fusion	This study
pDM4-P_{hcp1}::*lacZ*	pDM4 derivative for *hcp1* promoter fusion	This study
pDM4-P_{tae4}::*lacZ*	pDM4 derivative for *tae4* promoter fusion	This study
pDM4-P_{vgrG}::*lacZ*	pDM4 derivative for *vgrG* promoter fusion	This study
pKT100	Cloning vector, p15A replicon, Km^r	7
pKT100-*hcp1*-VSVG	pKT100 expressing *hcp1* with a C-terminal VSVG tag	This study

pBBR1MCS1	Cloning vector containing REP, Cm^r	8
pBBR1MCS1-*hns*	*hns* cloned into pBBR1MCS1 for complementation	This study
pBBR1MCS1-*fur*	*fur* cloned into pBBR1MCS1 for complementation	This study
pBBR1MCS1-*adrA*	pBBR1MCS1 expressing *adrA*	This study
pBBR1MCS1-*STM3611*	pBBR1MCS1 expressing *STM3611*	This study
pBBR1MCS1-*yedQ*	pBBR1MCS1 expressing *yedQ*	This study
pBBR1MCS1-FLAG-*hns*	pBBR1MCS1 expressing FLAG-H-NS	This study
pET-28a	Expression vector with N-terminal hexahistidine affinity tag, Km^r	Novagen
pET28a-*ycgR*	pET-28a expressing *ycgR*	This study
pET28a-*invF*	pET-28a expressing *invF*	1
pET28a-*stpA*	pET-28a expressing *stpA*	This study
pET28a-$stpA^{F98Y}$	pET-28a expressing $stpA^{F98Y}$	This study
pET28a-*hns*	pET-28a expressing *hns*	This study
pET28a-hns_{Ctd}	pET-28a expressing hns_{Ctd}	This study
pET28a-hns^{Y99A}	pET-28a expressing hns^{Y99A}	This study
pET28a-hns^{D101A}	pET-28a expressing hns^{D101A}	This study
pET28a-hns^{K107A}	pET-28a expressing hns^{K107A}	This study
pET28a-hns^{T115A}	pET-28a expressing hns^{T115A}	This study

***Km^r and Cm^r represent resistance to kanamycin and chloramphenicol, respectively.**

Table S2. Primers used in this study.

Primiers	5'-3' sequence*	
Δ*fur*-sg20-F	AGCTAGCTCAGTCCTAGGTATAATACTAGTCATCTTATCTGCCTTGATTGGTTTTAGAGCTAGAAATAGC	To generate pTargetF1-Δ*fur*
Δ*fur*-sg20-R	CCGAGTCGGTGCTTTTTTTGAAGCCACGATTGTGGGTCA	
Δ*fur*-up-F	TGCGGTATTGTTGTCAGTCA	
Δ*fur*-up-R	AAATCTTCCAGTTGCTTACCTTAAGTGCTTCGCTCATTGTAGTA	
Δ*fur*-down-F	TGACTGACAACAATACCGCATCACAGCCTCTATCTTTACGG	
Δ*fur*-down-R	GGTAATAGATCTAAGCTTCTGCAGGTCGACCGGACATCGGCATAGTTTT	
Δ*hns*-sg20-F	AGCTAGCTCAGTCCTAGGTATAATACTAGTAAGCGCTGCTGCTGCTGAAGGTTTTAGAGCTAGAAATAGC	To generate pTargetF1-Δ*hns*
Δ*hns*-sg20-R	GGGTGATGATAAGCATTGTTGTTCAAAAAAAGCACCGACTCGG	
Δ*hns*-up-F	CCGAGTCGGTGCTTTTTTTGAACAACAATGCTTATCATCACCC	
Δ*hns*-up-R	AAATCTTCCAGTTGCTTACCTTAAGTGCTTCGCTCATTGTAGTA	
Δ*hns*-down-F	TACTACAATGAGCGAAGCACTTAAGGTAAGCAACTGGAAGATTT	
Δ*hns*-down-R	GGTAATAGATCTAAGCTTCTGCAGGTCGACTTGTCGGGCGTTATGTGT	
Δ*clpV*-sg20-F	AGCTAGCTCAGTCCTAGGTATAATACTAGTTGTTGCAGATTGCCGAACCGGTTTTAGAGCTAGAAATAGC	To generate pTargetF1-Δ*clpV*
Δ*clpV*-sg20-R	GGGTGATGATAAGCATTGTTGTTCAAAAAAAGCACCGACTCGG	
Δ*clpV*-up-F	CCGAGTCGGTGCTTTTTTTGAACTGCGACTGTATTTTCCGTA	
Δ*clpV*-up-R	GTCGTGGCAGAATGTGCTGGGATTAGAGCGTAGTTTGC	
Δ*clpV*-down-F	GCAAACTACGCTCTAATCCCAGCACATTCTGCCACGAC	
Δ*clpV*-down-R	GGTAATAGATCTAAGCTTCTGCAGGTCGACTCAGCGTATTCGGCACAG	
hns(K107A)-sg20-F	AGCTAGCTCAGTCCTAGGTATAATACTAGTTGTATCCGCTTCTGTTCGCCGTTTTAGAGCTAGAAATAGC	To generate pTargetF1-*hns*(K107A)
hns(K107A)-sg20-R	GCCAAAAAGTAAAGTCACCAGTTCAAAAAAAGCACCGACTCGG	
hns(K107A)-up-F	CCGAGTCGGTGCTTTTTTTGAACTGGTGACTTTACTTTTTGGC	
hns(K107A)-up-R	TATCCGCAGACTTTAGCCATCGCCTGTTCAACCGCT	
hns(K107A)-down-F	AGCGGTTGAACAGGCGATGGCTAAAGTCTGCGGATA	
hns(K107A)-down-R	GGTAATAGATCTAAGCTTCTGCAGGTCGACAAGTGATT	

	GCTGAGCCGAT	
FLAG-*hns*-sg20-F	AGCTAGCTCAGTCCTAGGTATAATACTAGTCATCCGTACTCTTCGTGCGCGTTTTAGAGCTAGAAATAGC	To generate pTargetF1-FLAG-*hns*
FLAG-*hns*-sg20-R	TGCAATGAGATCGTATTTCATCTTCAAAAAAAGCACCGACTCGG	
FLAG-*hns*-up-F	CCGAGTCGGTGCTTTTTTTGAATGCAATGAGATCGTATTTCATC	
FLAG-*hns*-up-R	CTTATCGTCGTCCTTGTAATCCATTGTAGTAATCTCAAACTTATA	
FLAG-*hns*-down-F	ATGGATTACAAGGACGACGATAAGATGAGCGAAGCACTTAAAATTCT	
FLAG-*hns*-down-R	GGTAATAGATCTAAGCTTCTGCAGGTCGACTTAGCGGCAGCCATGCTATTCA	
P_{clpV}::*lacZ*-F	ACGCGTCGACATGTTGCTCAAAAAGCGGAA	To generate pDM4-P_{clpV}::lacZ
P_{clpV}::*lacZ*-R	CTAGTCTAGAAGTTTCCATGTGTTATGCCTTGT	
P_{hcp1}::*lacZ*-F	ACGCGTCGACTCGCTATTGGGTTTCGTGTA	To generate pDM4-P_{hcp1}::lacZ
P_{hcp1}::*lacZ*-R	CTAGTCTAGAGTCATAAGCCATTTTTATATCCTTA	
P_{tae4}::*lacZ*-F	ACGCGTCGACGATTGTCGCCGTGGTTTC	To generate pDM4-P_{tae4}::lacZ
P_{tae4}::*lacZ*-R	CTAGTCTAGATCTGTTCATTTTGCTTAACCTCTA	
P_{vgrG}::*lacZ*-F	ACGCGTCGACTGGTGAAAAGACATTTACGGTT	To generate pDM4-P_{vgrG}::lacZ
P_{vgrG}::*lacZ*-R	CTAGTCTAGATACAAAACTCATGGTAGTGACTCCT	
hcp1-F	GACGACAAGCTTACTAGTCTGCAGGGATCCATGGCTTATGACATTTTTTTGAA	To generate pKT100-*hcp1*-VSVG
hcp1-VSVG-R	ATCTTAGTTACTTAGGTACCCGGGGTCGACTTATTTTCCTAATCTATTCATTTCAATATCTGTATAAATTTCTTTGTTGGCCTTGAA	
C*hns*-F	GACGGTATCGATAAGCTTGATATCGAATTCATGAGCGAAGCACTTAAAATTCT	To generate pBBR1MCS1-*hns*
C*hns*-R	GGTGGCGGCCGCTCTAGAACTAGTGGATCCTTATTCCTTGATCAGGAAATCTTC	
C*fur*-F	GACGGTATCGATAAGCTTGATATCGAATTCATGACTGACAACAATACCGCA	To generate pBBR1MCS1-*fur*
C*fur*-R	GGTGGCGGCCGCTCTAGAACTAGTGGATCCTTATTTAGTCGCGTCATCGTG	
adrA-F	GACGGTATCGATAAGCTTGATATCGAATTCATGTTCCCAAAAATAATGAATGAT	To generate pBBR1MCS1-*adrA* *and* pBBR1MCS5-*adrA*
adrA-R	GGTGGCGGCCGCTCTAGAACTAGTGGATCCTCATGCCGCCACTTCG	
STM3611-F	GACGGTATCGATAAGCTTGATATCGAATTCATGATAAAGCAGGTTATCCAGC	To generate pBBR1MCS1-*STM3611* *and* pBBR1MCS5-*STM3611*
STM3611-R	GGTGGCGGCCGCTCTAGAACTAGTGGATCCTTACAGGGTCAGAATCACCTCT	
FLAG-*hns*-F	GACGGTATCGATAAGCTTGATATCGAATTCATGGATTACAAGGACGACGATGACAAGAGCGAAGCACTTAAAATTCT	To generate pBBR1MCS1-FLAG-*hns*

FLAG-*hns*-R	GGTGGCGGCCGCTCTAGAACTAGTGGATCCTTATTCCTTGATCAGGAAATCTTC	
ycgR-F	ACTGGTGGACAGCAAATGGGTCGCGGATCCGTGAGTGGTTACAATGAGCAGTTCC	To generate pET28a-*ycgR*
ycgR-R	GTGGTGGTGCTCGAGTGCGGCCGCAAGCTTTTATTCTCGCACTTTATTCGCTCTT	
stpA-F	ACTGGTGGACAGCAAATGGGTCGCGGATCCATGAATTTGATGTTACAGAACTTAA	To generate pET28a-*stpA*
stpA-R	GTGGTGGTGCTCGAGTGCGGCCGCAAGCTTTTAGATTAAGAAATCATCCAGAGAT	
stpA^{F98Y}-up-F	TCTTATTGTGCTGAAATATTCACTT	To generate pET28a-*stpA*^{F98Y}
stpA^{F98Y}-up-R	TCTTCGCCATTGAAATCAGTATAACGATATTTTGCCGGAC	
stpA^{F98Y}-down-F	GTCCGGCAAAATATCGTTATACTGATTTCAATGGCGAAGA	
stpA^{F98Y}-down-R	TTTCCTCATTAGCGGTTTTG	
hns-F	ACTGGTGGACAGCAAATGGGTCGCGGATCCATGAGCGAAGCACTTAAAATTCT	To generate pET28a-*hns*
hns-R	GTGGTGGTGCTCGAGTGCGGCCGCAAGCTTTTATTCCTTGATCAGGAAATCTTC	
hns$_{Ctd}$-F	ACTGGTGGACAGCAAATGGGTCGCGGATCCATGGCAGCTCGTCCGGCTAA	To generate pET28a-*hns*$_{Ctd}$
hns$_{Ctd}$-R	GTGGTGGTGCTCGAGTGCGGCCGCAAGCTTTTATTCCTTGATCAGGAAATCTTC	
hns^{Y99A}-up-F	TGATAAAATGTGACCTGACTCCTA	To generate pET28a-*hns*^{Y99A}
hns^{Y99A}-up-R	TAGTTTCACCGTTTTCGTCAACAGCGCTATATTTAGCCGGAC	
hns^{Y99A}-down-F	GTCCGGCTAAATATAGCGCTGTTGACGAAAACGGTGAAACTA	
hns^{Y99A}-down-R	ATTGTCGGGCGTTATGTGTT	
hns^{D101A}-up-F	CGGTGAGTATCCCCCCTG	To generate pET28a-*hns*^{D101A}
hns^{D101A}-up-R	CAGGTTTTAGTTTCACCGTTTTCGGCAACATAGCTATATTTAGCCG	
hns^{D101A}-down-F	CGGCTAAATATAGCTATGTTGCCGAAAACGGTGAAACTAAAACCTG	
hns^{D101A}-down-R	ATTGTCGGGCGTTATGTGTT	
hns^{K107A}-up-F	CAGACGGTGAGTATCCCCC	To generate pET28a-*hns*^{K107A}
hns^{K107A}-up-R	CCCTGGCCAGTCCAGGTTGCAGTTTCACCGTTTTCG	
hns^{K107A}-down-F	CGAAAACGGTGAAACTGCAACCTGGACTGGCCAGGG	
hns^{K107A}-down-R	AAAAACCAAAAGCGGATGTC	
hns^{T115A}-up-F	TCTTTTTTGTGCGGTGCCT	To generate pET28a-*hns*^{T115A}
hns^{T115A}-up-R	TGCTTTTTTGATTACAGCCGGTGCACGACCCTGGCCA	
hns^{T115A}-down-F	TGGCCAGGGTCGTGCACCGGCTGTAATCAAAAAAGCA	
hns^{T115A}-down-R	TGGTTGGCGTGGTTGAAAA	

16S-F<br>16S-R	GAAGGTGTTGTGGTTAATA<br>AGTAATTCCGATTAACGCTT	qRT-PCR
clpV-RT-F<br>*clpV*-RT-R	AAAAAATGGGGATTCAGTC<br>CCTGCCACTCCCTTAGC	qRT-PCR
hcp1-RT-F<br>*hcp1*-RT-R	GGATGACAAACACAAAAATGAAA<br>ACAATCAGGTCGGTGAAGGTA	qRT-PCR
tae4-RT-F<br>*tae4*-RT-R	ATTCCGTCGCTGATGTG<br>TCAGGCTTGCCCATAGTA	qRT-PCR
vgrG-RT-F<br>*vgrG*-RT-R	AAGAGTGAAGGCGAGATGCT<br>AAACGGGTGGAAAAGGTAAA	qRT-PCR
ssrB-RT-F<br>*ssrB*-RT-R	TCCTTGATCTTAGTCTACCTGG<br>CTGCTTTTTAAAACATAGCCAT	qRT-PCR
sseA-RT-F<br>*sseA*-RT-R	TAAGGTGAGTCAACAGCTTGC<br>TTGTTTTTCCTGACGGTATCT	qRT-PCR
rmbA-RT-F<br>*rmbA*-RT-R	TGACAGCGCAGTACGGGT<br>CGTCGAAGATTCGGGAAA	qRT-PCR
misL-RT-F<br>*misL*-RT-R	CAGCATCCTCCTCCAGAAC<br>TCGCCCGCCATAGTAATA	qRT-PCR
slsA-RT-F<br>*slsA*-RT-R	CTGGCTAAGATCGCCACCTT<br>CGCTTTTACCGCTTGTACAAAA	qRT-PCR
pipA-RT-F<br>*pipA*-RT-R	ACTTCAAACTTTCAGAAGGCAG<br>GGTATATTCGACAACAGGGC	qRT-PCR
pipB-RT-F<br>*pipB*-RT-R	AGTAAACATAATATACAACAGCCT<br>AACACAAATTTGCACCG	qRT-PCR
stpA-RT-F<br>*stpA*-RT-R	CTTAAATAATATCCGCACGC<br>CCATTGAAATCAGTAAAACG	qRT-PCR
ropS-RT-F<br>*ropS*-RT-R	ACAATCGAACGGGCGATC<br>CGGGGTGTCTACCGAGGTA	qRT-PCR
phoP-RT-F<br>*phoP*-RT-R	GGCGCAGCAGTGATGTTT<br>AAGCGTTTCCATAATGGTGTAT	qRT-PCR
phoQ-RT-F<br>*phoQ*-RT-R	TTCCATGAAATTGAAACCAA<br>TATACGAACCAGCTCCACAC	qRT-PCR
fimA-RT-F<br>*fimA*-RT-R	GTGCCTTTCTCCATCGTCC<br>CAGCGTATTGGTGCCTTCA	qRT-PCR
yaiU-RT-F<br>*yaiU*-RT-R	CTTTTGGCACAGGTCACTTT<br>TATCATCACCATCAGCATCG	qRT-PCR
clpV-ChIP-F<br>*clpV*-ChIP-R	TCTGTATCATTGTTTTGTTTCGATA<br>AGTTTCCATGTGTTATGCCTTG	ChIP-qPCR
vgrG-ChIP-F<br>*vgrG*-ChIP-R	TATTTTCATGGCTCTTGTCAGCAAT<br>GGTAGTGACTCCTTTAATACCGGAT	ChIP-qPCR
ssrAB-ChIP-F	TAATTGTAGTCATCGACTGGGTTAT	ChIP-qPCR

ssrAB-ChIP-R	AATGCTTCCCTCCAGTTGC	
sseA-ChIP-F	TAGTACGTGAGGTTTGACTCGC	ChIP-qPCR
sseA-ChIP-R	TCCCCTCCATATACACGATAGA	
rmbA-ChIP-F	TGTCATGGTTCCTTAAAGCTGTATT	ChIP-qPCR
rmbA-ChIP-R	CCTGAACGACTCCTGTCGATG	
slsA-ChIP-F	CTGTCATCAGCTTAAAAACGG	ChIP-qPCR
slsA-ChIP-R	TTAATTCATTCCTTGTTGTTGCCTT	
pipA-ChIP-F	GCCCCTAAGTTTCATTATAAATA	ChIP-qPCR
pipA-ChIP-R	AACTTCTTATTTCCTGACTACAATT	
pipB-ChIP-F	CTACTCATACTCAACCAAAGCTCTA	ChIP-qPCR
pipB-ChIP-R	TTTGATTCCTTCTTATGGAAGTG	
fimA-ChIP-F	AGTTTGCGGCTATTTTTTATTT	ChIP-qPCR
fimA-ChIP-R	GGATTTCCCTTGAATTACACAC	
yaiU-ChIP-F	AAGTAAATGTGGGTTTTAACATTATTCA	ChIP-qPCR
yaiU-ChIP-R	TTGTCATCCCTCCTCAGGGAC	
P_{clpV}-EMSA-F	TTTTTATCTTGCTGTTCATTGATTA	EMSA
P_{clpV}-EMSA-R	TTCCATGTGTTATGCCTTGTG	
P_{clpV}-EMSA-CK-F	TGTTTGACAAGGGTGGGATG	To generate fragment of negative control for EMSA
P_{clpV}-EMSA-CK-R	CAGCGGCAGGTAAGGGAT	
P_{vgrG}-EMSA-F	TATTGTTATGTTCATTTCCTTATTTA	EMSA
P_{vgrG}-EMSA-R	CAAAACTCATGGTAGTGACTCC	
P_{vgrG}-EMSA-CK-F	TATTGTTATGTTCATTTCCTTATTTA	To generate fragment of negative control for EMSA
P_{vgrG}-EMSA-CK-R	CAAAACTCATGGTAGTGACTCC	
P_{fimA}-EMSA-F	CGGCTATTTTTTATTTAGCGAAA	EMSA
P_{fimA}-EMSA-R	ATGTTTCATGGATTTCCCTTGA	
P_{fimA}-EMSA-CK-F	TACGGCGATTGGTAATACGACT	To generate fragment of negative control for EMSA
P_{fimA}-EMSA-CK-R	TGCCTTATAGCGTGCGGTAA	
P_{yaiU}-EMSA-F	TTAATACCTCAGGCACTATATCATTTTA	EMSA
P_{yaiU}-EMSA-F	CAAGAAGGATTATGCGGCG	
P_{yaiU}-EMSA-CK-F	GGTTCAGATTATATCCTGGATAGTGC	To generate fragment of negative control for EMSA
P_{yaiU}-EMSA-CK-R	CATTATCAAGTGTAAGGTTCAGTTCGT	
P_{ssrAB}-EMSA-F	TACAATATCAGGATGCTGTCTACA	EMSA
P_{ssrAB}-EMSA-R	TAATCGATGGTGTTATCATTAGG	
P_{ssrAB}-EMSA-CK-F	AACCCATGCCGGGTTTTACT	To generate fragment of negative control for EMSA
P_{ssrAB}-EMSA-CK-R	ATCCATCATGCAGCGTTACATT	
P_{sseA}-EMSA-F	AACATTATTTATTATCGTTACCATAAC	EMSA
P_{sseA}-EMSA-R	GCTATTTTACTTGCCATTTTGA	
P_{sseA}-EMSA-CK-F	GCTGCGTTTAGTGAATATCGT	To generate fragment of negative control for EMSA
P_{sseA}-EMSA-CK-R	TATCTCCACCGGGGCTT	
P_{rmbA}-EMSA-F	ATAGTGACGAAGGCAGCAGA	EMSA
P_{rmbA}-EMSA-R	AAACTCCTGGAAAGACCTAAAAT	

P$_{rmbA}$-EMSA-CK-F<br>P$_{rmbA}$-EMSA-CK-R	GCGGTTTTATCTTTGTTGAT<br>TTCGTCCACGTTTACTGTTG	To generate fragment of negative control for EMSA
P$_{slsA}$-EMSA-F<br>P$_{slsA}$-EMSA-R	TTTCAATCAATAATCCTCTC<br>TGATAGTCACTATCAGTGTTAAAGTCA	EMSA
P$_{slsA}$-EMSA-CK-F<br>P$_{slsA}$-EMSA-CK-R	GGGTACAAGGTATTTGCGG<br>AATCCAGCAGTTCATTATTTTT	To generate fragment of negative control for EMSA
P$_{pipA}$-EMSA-F<br>P$_{pipA}$-EMSA-R	AGGTAGTCAACATACCCCTACTC<br>CATACAGGTAACGCTATGATTCA	EMSA
P$_{pipA}$-EMSA-CK-F<br>P$_{pipA}$-EMSA-CK-R	ACAGATTAATACCTCAAAGCGG<br>GGATAGTTCATCGTAGCATTCG	To generate fragment of negative control for EMSA
P$_{pipB}$-EMSA-F<br>P$_{pipB}$-EMSA-R	TTTATTCAATGATGATGAAGCGT<br>TTCTTATGGAAGTGAGCCGAC	EMSA
P$_{pipB}$-EMSA-CK-F<br>P$_{pipB}$-EMSA-CK-R	TTTGGAATGTATGTCGAATGTTA<br>CAAGTCAGGTCTGCGTGAGT	To generate fragment of negative control for EMSA

***Underlined sites indicate restriction enzyme cutting sites added for cloning.**

Supplementary References

1. Li, S. et al. Autoinducer-2 and bile salts induce c-di-GMP synthesis to repress the T3SS via a T3SS chaperone. *Nat Commun*. **13,** 6684 (2022).
2. Simon, R., Priefer, U. & Puhler, A. A broad host range mobilization system for in vivo genetic engineering: transposon mutagenesis in gram negative bacteria. *Nat. Biotechnol.* **1,** 784–791 (1983).
3. Jiang, Y. et al. Multigene editing in the *Escherichia coli* genome via the CRISPR-Cas9 system. *Appl. Environ. Microbiol.* **81,** 2506–2514 (2015).
4. Zhang, L. et al. The catabolite repressor/activator Cra is a bridge connecting carbon metabolism and host colonization in the plant drought resistance-promoting bacterium *Pantoea alhagi* LTYR-11Z. *Appl. Environ. Microbiol.* **84,** e00054-18 (2018).
5. Schäfer, A. et al. Small mobilizable multi-purpose cloning vectors derived from the Escherichia coli plasmids pK18 and pK19: selection of defined deletions in the chromosome of *Corynebacterium glutamicum. Gene* **145,** 69–73 (1994).
6. Zhao, Y. & Shao, F. The NAIP-NLRC4 inflammasome in innate immune detection of bacterial flagellin and type III secretion apparatus. *Immunol. Rev.* **265,** 85–102 (2015).
7. Hu, Y. et al. OmpR positively regulates urease expression to enhance acid survival of *Yersinia pseudotuberculosis. Microbiology* **155,** 2522–2531 (2009).
8. Kovach, M. E. et al. Four new derivatives of the broad-host-range cloning vector pBBR1MCS, carrying different antibiotic-resistance cassettes. *Gene* **166**, 175–176 (1995).
