# Supplementary material for: c-di-GMP inhibits the DNA binding activity of H-NS in Salmonella
Source: Nat Commun. 2023 Nov 18;14:7502. doi: 10.1038/s41467-023-43442-5 (PMC10657408; doi:10.1038/s41467-023-43442-5)
Supplement: Supplementary file 3 — Description of Additional Supplementary Files [file 41467_2023_43442_MOESM3_ESM.pdf]

Description of Additional Supplementary Files:

Supplementary Data 1: Differentially expressed genes (absolute $\log_2$ fold change > 1, Benjamini–Hochberg adjusted $p < 0.05$) in Δhns or the wild type (WT) overexpressing adrA compared to the WT based on RNASeq.
