# Supplementary material for: c-di-GMP inhibits the DNA binding activity of H-NS in Salmonella
Source: Nat Commun. 2023 Nov 18;14:7502. doi: 10.1038/s41467-023-43442-5 (PMC10657408; doi:10.1038/s41467-023-43442-5)
Supplement: Supplementary file 5 — Reporting Summary [file 41467_2023_43442_MOESM5_ESM.pdf]

nature portfolio

Corresponding author(s): Lei Zhang and Xihui Shen

Last updated by author(s): Nov 5, 2023

Reporting Summary

Nature Portfolio wishes to improve the reproducibility of the work that we publish. This form provides structure for consistency and transparency in reporting. For further information on Nature Portfolio policies, see our Editorial Policies and the Editorial Policy Checklist.

Statistics

For all statistical analyses, confirm that the following items are present in the figure legend, table legend, main text, or Methods section.

n/a	Confirmed	
☐	☒	The exact sample size (*n*) for each experimental group/condition, given as a discrete number and unit of measurement
☐	☒	A statement on whether measurements were taken from distinct samples or whether the same sample was measured repeatedly
☐	☒	The statistical test(s) used AND whether they are one- or two-sided<br>*Only common tests should be described solely by name; describe more complex techniques in the Methods section.*
☒	☐	A description of all covariates tested
☐	☒	A description of any assumptions or corrections, such as tests of normality and adjustment for multiple comparisons
☐	☒	A full description of the statistical parameters including central tendency (e.g. means) or other basic estimates (e.g. regression coefficient) AND variation (e.g. standard deviation) or associated estimates of uncertainty (e.g. confidence intervals)
☐	☒	For null hypothesis testing, the test statistic (e.g. *F*, *t*, *r*) with confidence intervals, effect sizes, degrees of freedom and *P* value noted<br>*Give P values as exact values whenever suitable.*
☒	☐	For Bayesian analysis, information on the choice of priors and Markov chain Monte Carlo settings
☒	☐	For hierarchical and complex designs, identification of the appropriate level for tests and full reporting of outcomes
☒	☐	Estimates of effect sizes (e.g. Cohen's *d*, Pearson's *r*), indicating how they were calculated

Our web collection on statistics for biologists contains articles on many of the points above.

Software and code

Policy information about availability of computer code

Data collection	TA NANO-ITC SV microcalorimeter, ITCRun software v3.80, Tanon 5200Multi Chemiluminescent Imaging System, AB SCIEX QTRAP 6500+ LC-MS/MS System, AKTA Purifier FPLC system, LightCycler 96 thermocycler, Biotek Epoch Microplate Reader
Data analysis	Microsoft Excel 2019, Prism Graphpad 8.0, Primer premier 5.0, Tanon gel analysis 2.30, NanoAnalyze_3.4, POCASA v1.1, AutoDock4, AutoDock Vina v1.1.2, PyMOL v2.5.2, LigPlot+ v2.2.4, ImageJ v1.52a, Bowtie v2.3.2, FeatureCounts v1.6.0, DESeq2 R package v1.12.4

For manuscripts utilizing custom algorithms or software that are central to the research but not yet described in published literature, software must be made available to editors and reviewers. We strongly encourage code deposition in a community repository (e.g. GitHub). See the Nature Portfolio guidelines for submitting code & software for further information.

Data

Policy information about availability of data

All manuscripts must include a data availability statement. This statement should provide the following information, where applicable:

- Accession codes, unique identifiers, or web links for publicly available datasets
- A description of any restrictions on data availability
- For clinical datasets or third party data, please ensure that the statement adheres to our policy

The protein 3D coordinate data used in this study are available in the PDB database under the accession code 2L93 (https://www.rcsb.org/structure/2L93). The structure of c-di-GMP used in this study is available from the crystal structure of the c-di-GMP-MapZ complex in the PDB database under the accession code 2L74 (https://www.rcsb.org/structure/2L74). The S. Typhimurium LT2 reference genome used in this study is available in the NCBI nucleotide database under the

accession numbers NC_003197 (https://www.ncbi.nlm.nih.gov/nuccore/NC_003197) and NC_003277 (https://www.ncbi.nlm.nih.gov/nuccore/NC_003277). The RNA-seq data generated in this study have been deposited in the NCBI BioProject database under the accession number PRJNA975738 (https://www.ncbi.nlm.nih.gov/bioproject/?term=PRJNA975738). All the other data that support the findings of this study are available within the paper and its Supplementary Information and Supplementary Data. The source data underlying Figs. 1, 2a–c, f, 3, 4 and 5c–e and Supplementary Figs. 1–7, 9a, 10b and 11–17 are provided as a Source Data file.

Research involving human participants, their data, or biological material

Policy information about studies with human participants or human data. See also policy information about sex, gender (identity/presentation), and sexual orientation and race, ethnicity and racism.

Reporting on sex and gender	N/A
Reporting on race, ethnicity, or other socially relevant groupings	N/A
Population characteristics	N/A
Recruitment	N/A
Ethics oversight	N/A

Note that full information on the approval of the study protocol must also be provided in the manuscript.

Field-specific reporting

Please select the one below that is the best fit for your research. If you are not sure, read the appropriate sections before making your selection.

- [x] Life sciences
- [ ] Behavioural & social sciences
- [ ] Ecological, evolutionary & environmental sciences

For a reference copy of the document with all sections, see nature.com/documents/nr-reporting-summary-flat.pdf

Life sciences study design

All studies must disclose on these points even when the disclosure is negative.

Sample size	Sample sizes were determined without statistical measures, but based on widely used sizes in relevant publications (Zhang et al. Nat. Commun. 2020, 11:5371; Gan et al. Nat Microbiol. 2019, 4:134-143; Lin et al. Nat. Commun. 2017, 8:14888; Zheng et al. Mol. Microbiol. 2013, 89:403-419; Li et al. Nat. Commun. 2022, 13:6684) to ensure that it will be appropriate for statistical analysis.
Data exclusions	No data were excluded from the analyses.
Replication	All experiments were performed for at least 3 times. All the attempts at replication were successful.
Randomization	No experimental groups or control groups were subjectively chosen and there are no covariates to control for as experiments were done in isogenic strains. No experiments required randomization.
Blinding	Investigators were not blinded during data collection or analysis since there was not group allocation.

Reporting for specific materials, systems and methods

We require information from authors about some types of materials, experimental systems and methods used in many studies. Here, indicate whether each material, system or method listed is relevant to your study. If you are not sure if a list item applies to your research, read the appropriate section before selecting a response.

Materials & experimental systems

n/a	Involved in the study	
[ ]	[x]	Antibodies
[x]	[ ]	Eukaryotic cell lines
[x]	[ ]	Palaeontology and archaeology
[x]	[ ]	Animals and other organisms
[x]	[ ]	Clinical data
[x]	[ ]	Dual use research of concern
[x]	[ ]	Plants

Methods

n/a	Involved in the study	
[x]	[ ]	ChIP-seq
[x]	[ ]	Flow cytometry
[x]	[ ]	MRI-based neuroimaging

Antibodies

Antibodies used	Rabbit anti-ICDH is a kind gift from Dr. ZQ. Luo at Purdue University and was described in: Xu, L. et al. PLoS Pathog. 6, e1000822 (2010). Purified ICDH was used to inject rabbits and the antisera were affinity purified using Protein A Sepharose (GE Healthcare). Commercial antibodies used are listed as below: Mouse anti-VSVG (Abways, Shanghai, China, clone 4A10, cat# AB0063); Mouse anti-FLAG (Abways, Shanghai, China, clone 5C3, cat# AB0028); Mouse anti-His (Abways, Shanghai, China, clone 3B5, cat# AB0002); Streptavidin-horseradish peroxidase (Thermo Scientific, cat# 21126); Goat anti-Mouse horseradish peroxidase-conjugated secondary antibodies (DIYIBIO, China, cat# DY60203); Goat anti-Rabbit horseradish peroxidase-conjugated secondary antibodies (DIYIBIO, China, cat# DY60202).
Validation	The homemade antibody Rabbit anti-ICDH has been validated by our lab using appropriate positive and negative controls in previous studies (Li et al. Nat. Commun. 2022, 13:6684; Zhu et al., Proc. Natl. Acad. Sci. U S A. 2021, 118(42):e2103526118).<br>The antibodies obtained commercially have been validated by the manufacturers.<br>Antibody, catalogue number, manufacturer information for commercial antibodies:<br>-Mouse anti-VSVG (Abways, Shanghai, China, cat# AB0063): http://www.abways.com/showproduct.asp?cid=AB0063<br>-Mouse anti-FLAG (Abways, Shanghai, China, cat# AB0028): http://www.abways.com/showproduct.asp?cid=AB0028<br>-Mouse anti-His (Abways, Shanghai, China, cat# AB0002): http://www.abways.com/showproduct.asp?cid=AB0002<br>-Streptavidin-horseradish peroxidase (Thermo Scientific, cat# 21126) : https://www.thermofisher.cn/order/catalog/product/21126<br>-Goat anti-Mouse horseradish peroxidase-conjugated secondary antibodies (DIYIBIO, China, cat# DY60203): https://www.deeyeebio.com/list_51/243.html<br>-Goat anti-Rabbit horseradish peroxidase-conjugated secondary antibodies (DIYIBIO, China, cat# DY60202): https://www.deeyeebio.com/list_51/242.html
